# The role of lipid traits in mediating the effect of body mass index on serum urate

**DOI:** 10.3389/fendo.2022.938891

**Published:** 2022-09-23

**Authors:** Liuqing Peng, Jiarui Jing, Simin He, Juping Wang, Xue Gao, Tong Wang

**Affiliations:** Department of Health Statistics and Epidemiology, School of Public Health, Shanxi Medical University, Taiyuan, China

**Keywords:** body mass index (BMI), serum urate, lipid traits, mediation analysis, Mendelian randomization

## Abstract

**Objective:**

To explore whether total cholesterol (TC), high-density lipoprotein (HDL), low-density lipoprotein (LDL), and triglyceride (TG) are mediators in the pathway of body mass index (BMI) on serum urate and determine the proportion of the mediation effect.

**Methods:**

This study used observational and two-sample Mendelian randomization (MR) analyses to explore the mediation effects of TC, HDL, LDL, and TG in the pathway of BMI on serum urate. We determined the size and the extent to which these lipids mediate any effect of BMI on serum urate.

**Results:**

Observational analysis results showed that HDL and TG can partially explain the association of BMI on serum urate, and the proportion of mediation effect was 10.2% and 8.9%, respectively. MR results demonstrated that TG has a causal effect on serum urate (*β* = 0.22, 95% CI: 0.15, 0.29; *p* = 2.28×10^–10.^) and its proportion of mediation effect was 14.1%. TC, HDL, and LDL are not the mediators in the pathway of BMI on serum urate in MR estimates.

**Conclusion:**

To a certain extent, TG mediates the effect of BMI on serum urate, and the risk of gout may be reduced by controlling both BMI and TG.

## Introduction

Gout is a chronic disease caused by the deposition of monosodium urate (MSU) crystals (1). The global prevalence of gout is on the rise: the result of the Global Burden of Disease Study in 2017 showed that the age-standardized prevalence rates of gout in men and women in 1990 were 747.48/100,000 and 233.52/100,000, but the number had risen to 790.90/100,000 and 253.49/100,000 in 2017, respectively (2). In 2010, the global disability-adjusted life years for gout were 11,400, an increase of 38,000 compared to those in 1990 (3). Now, gout has become the most common inflammatory arthritis in developed countries (4). Elevated serum urate is the direct cause of MSU crystal deposition and the development of gout (5). However, the proportion of patients who initiate and continue urate-lowering therapy is very low, ranging from 10% to 46%, worldwide (2, 6). Studies have suggested that mature adipocytes and adipose tissue produce and secrete urate. Moreover, obesity increases the expression of xanthine oxidoreductase mRNA and promotes serum urate secretion in adipose tissue (7, 8). Researchers have shown that obesity is an important risk factor for hyperuricemia (5, 9). Dalbeth and colleagues found that people with a mean body mass index (BMI) of 30.8 kg/m^2^ had a higher level of serum urate than those with a mean BMI of 21.8 kg/m^2^ (10). Furthermore, studies suggested that greater BMI leads to higher levels of total cholesterol (TC), low-density lipoprotein (LDL), and triglyceride (TG) but lower high-density lipoprotein (HDL) levels (11). Moreover, hyperuricemia is usually related to lipid metabolism disorders (12). Thus, the association of BMI with serum urate may be mediated by these lipids. However, to our knowledge, there is no published literature about the mediation analysis of the above lipids in the pathway of BMI on serum urate.

In mediation analysis, three parameters are typically estimated: total effect (the effect of exposure on outcome through all potential pathways), direct effect (the effect of exposure on outcome when controlling for mediators), and indirect effect (the effect of exposure on outcome that acts through mediators).

**Figure 1:**
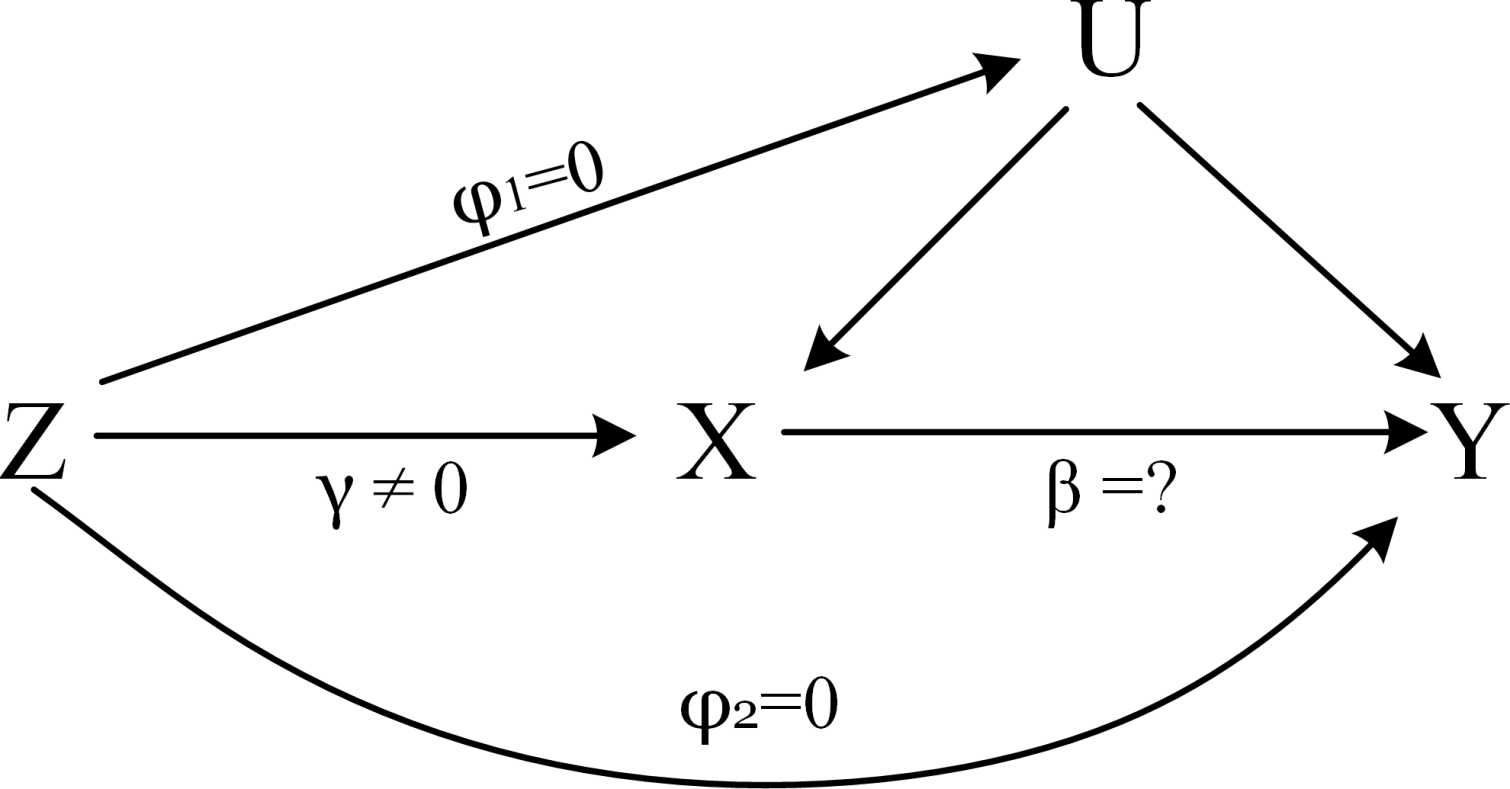
The core assumptions of MR. *Z* represents the genetic instrument (SNPs), *X* the exposure (BMI, TC, HDL, LDL, and TG), *Y* the outcome (serum urate), and *U* denotes the confounders of the relationship of *X*–*Y*. Assumptions: 1. Genetic variation is closely related to the exposure of interest, *γ* ≠ 0; 2. Genetic variation has nothing to do with confounding, *φ*_1_ = 0; 3. Genetic variation only affects the outcome through *X*, *φ*_2_ = 0.

Mendelian randomization (MR) such as the two-step Mendelian randomization (13) and multivariable Mendelian randomization (14) has been used to investigate mediation effects in recent years. MR uses genetic variants, normally single nucleotide polymorphisms (SNPs), as instrumental variables to estimate the causal effect of an exposure on an outcome free from bias due to unknown confounders and reverse causality (15). **Figure 1** describes the core assumptions of MR (16).

MR has previously been used to investigate the causal effects of BMI on serum urate, TC, HDL, LDL, and TG and also the effects of these four lipids on serum urate. Studies have shown the association of BMI, lipids, and serum urate (17–19), but their mediation effects are still to be determined. In this study, we investigated the role of TC, HDL, LDL, and TG in mediating the association of BMI with serum urate.

Due to the low rates of urate-lowering therapy in gout patient, understanding the changes of these four lipids in the risk of BMI on gout and preventing these risk factors are veryimportant public health aims and are primary prevention measures for higher-risk populations.

## Materials and methods

### Overall study design

We first established the association of BMI with serum urate and then explored the mediation effect of the lipid traits. The data analysis of this study was organized into two parts: part 1 involves observational analysis using the product of coefficients (20) of the data from the National Health and Nutrition Examination Survey (NHANES), and part 2 involves two-sample MR analysis with summary-level genetic data. This involved the two-step two-sample MR (13) and multivariable Mendelian randomization (14). We first used the two-step MR to identify which of the lipids are possible mediators. Then, multivariable MR was used to calculate the direct effect of BMI and mediators on serum urate in order to calculate the size and proportion of the mediation effect.

### Data sources

#### Observational analysis

NHANES is a population-based survey designed to collect information on the health and nutrition of the US household population. Since data collection for 2019–2020 has not been completed, the data from the NHANES 2017–2018 survey were selected for this study. In this study, we obtained 3,036 participants, of which 819 individuals were excluded due to missing data, and 2,217 were eventually included. We collected age, gender, education level (the highest level or level of education completed by adults 20 years and older), marital status, family annual income, place of birth, race, BMI, TC, HDL, LDL, TG, and serum urate. Among the 2,217 participants included, men accounted for 47.9%, and women accounted for 52.1%. The mean age (standard deviation) of the participants was 51.32 (17.65), 41.3% were under 60 years old, and 52.7% were over 60 years old. The average levels of BMI, TC, HDL, LDL, TG, and urate were 29.82 kg/m^2^ (7.49), 185.75 mg/dl (41.09), 53.87 mg/dl (15.53), 110.38 mg/dl (36.19), 107.38 mg/dl (63.66), and 5.53 mg/dl (1.50), respectively.

#### Two-sample MR analysis

Summary-level data were made publicly available and ethical approval was obtained in the original studies (**Table 1**). Summary-level data for BMI were derived from a recently published meta-analysis of GWAS, including 681,275 European descent individuals from the Genetic Investigation of Anthropometric Traits consortium (GIANT) (21). Genetic association with HDL (*N* = 403,943), LDL (*N* = 440,546), and TG (*N* = 441,016) were obtained from the UK Biobank (22). The average age of these participants was 56.9 (range: 39–73) years old and 54.2% were women. The mean values of the lipid concentrations (standard deviation) were as follows: HDL = 1.45 (0.38) mmol/L, LDL = 3.57 (0.87) mmol/L, and TG (median) = 1.50 (1.11) mmol/L. Summary-level data for TC were obtained from the Global Lipids Genetics Consortium (GLGC) involving 187,365 participants, and these participants were from multiple ethnic populations including European, East Asian, South Asian, and African (23). For serum urate, summary-level data were from the Global Urate Genetics Consortium genome-wide association study of serum urate involving 110,347 participants of European ancestry (24). The serum urate concentration ranged from 3.9 to 6.1 mg/dl (median = 5.2 mg/dl). All SNPs used in each two-sample MR analysis are provided in the **Supplementary File**.

**Table 1:** GWAS cohorts used in this study.

Phenotype	First author (year)	Sample size	Consortium
BMI	Yengo (2018)	681,275	GIANT
TC	Willer (2013)	187,365	GLGC
HDL	Richardson (2020)	403,943	UK Biobank
LDL	Richardson (2020)	440,546	UK Biobank
TG	Richardson (2020)	441,016	UK Biobank
Serum urate	Kottgen (2013)	110,347	GUGC

### Statistical analysis

#### Association of BMI on serum urate

In the observational analysis, we used multivariable linear regression to estimate the association of BMI with serum urate. We adjusted confounders including age, gender, annual family income, education, marital status, race, and place of birth (25–27).

In the two-sample MR analysis, the causal effects were investigated using the ratio method with standard errors derived using the delta method (28). We used the random-effects inverse variance weighted (IVW) method to pool MR estimates across individual SNPs (29).

#### Mediation by TC, HDL, LDL, and TG

In the observational mediation analysis, when investigating the degree to which the effects of BMI on serum urate are mediated through each lipid trait (TC, HDL, LDL, and TG) individually, we used the product of coefficients method to estimate the indirect effect (20). This involved first estimating the association of BMI on each lipid trait individually and then multiplying this with the direct effect of those lipid traits on serum urate after adjusting for BMI. We used multivariable linear regression to estimate the association of BMI with each lipid trait after adjusting for confounders (age, gender, annual family income, education, marital status, race, and place of birth). We then estimated the direct effect of each lipid trait on serum urate after adjusting for BMI, age, gender, annual family income, education, marital status, race, and place of birth. The two estimates were multiplied together to estimate the indirect effect with confidence intervals derived using the non-parametric bootstrap method (simulation times = 1,000). Finally, we estimated the proportion of the effect of BMI on serum urate that was mediated by each lipid trait by dividing the indirect effect by the total effect.

In the two-sample MR analysis, we used the two-step two-sample MR (13) to identify the potential mediator. The first step involved estimating the causal effects of BMI on TC, HDL, LDL, and TG, and the second step involved estimating the causal effects of each lipid trait on serum urate. Then, BMI and the mediators (the lipid trait was not only influenced by BMI but also affected by serum urate) determined by the above steps were simultaneously included in the multivariable Mendelian randomization model (14) to estimate their direct effects on serum urate. We estimated the indirect effect by multiplying the effect of BMI on mediators with the direct effect of mediators on serum urate. The proportion of mediation was calculated by dividing the indirect effect by the total effect.

In the selection of SNPs to address the potential weak instrument bias, we included SNPs that were genome-wide significantly ( *p<* 5×10^–8^). All *F* statistics used in MR were above 30, suggesting that a weak instrument bias was unlikely (more details seen in the **Supplementary File**). To further ensure the independence of the instruments, SNPs that were in linkage disequilibrium (LD) were excluded from the instrument variable set using the clumping algorithm (*r* ^2^ threshold > 0.01 and window size = 5,000 kb).

#### Sensitivity analysis

In the observational analysis, 819 individuals were excluded due to data missing, and the proportion of missing values was about 26%. To avoid possible selection bias, we imputed values for those subjects with missing data using the MissForest method (30). This method can successfully handle missing values in the range of 10%–30%, especially in datasets including different types of variables (30). We then performed the observational analysis again for 3,036 subjects with complete data.

In the two-sample MR, a range of sensitivity analyses was carried out, including MR-Egger, weighted median estimator (WME), and MR pleiotropy residual sum and outlier (MR-PRESSO) (31–33). MR-Egger provides a valid test of pleiotropy, and the estimated value of the intercept in MR-Egger can be interpreted as an estimate of the average pleiotropic effect across the SNPs. Moreover, the estimator of WME is consistent even when up to 50% of the information comes from invalid SNPs. MR-PRESSO was used to identify horizontal pleiotropic SNPs and then estimated the causal effect excluding these SNPs. Furthermore, mediation will be estimated with bias if serum urate has an effect on BMI or the four lipid traits (means reverse causality). We tested the reverse causal effect of BMI, TC, HDL, LDL, TG, and serum urate with each other using the IVW and MR-Egger methods.

In this study, the estimates were betas. To account for multiple testing, we employed a Bonferroni-corrected threshold of *p<*0.0056 (0.05/9 to correct for one exposure and four lipids in relation to serum urate). All statistical analysis was performed using R (version 4.1.0.) software. We used “TwoSampleMR (0.5.5)” package and “mediation” package to implement MR and observational mediation analyses.

## Results

### Observational association of BMI with mediators and outcome

After adjusting for age, gender, annual family income, education, marital status, race, and place of birth, the total effect of BMI on serum urate was 0.058 (95% CI: 0.051, 0.065; *p<* 2×10^–16^), on TC −0.22 (95% CI: −0.46, 0.015; *p*=0.66), on HDL −0.62 (95% CI: −0.69, −0.54; *p<* 2×10^–16^), on LDL 0.091 (95% CI: −0.11, 0.30; *p* =0.38), and on TG 1.55 (95% CI: 1.21, 1.89; *p<* 2×10^–16^).

### Observational mediation analysis

After adjusting for age, gender, annual family income, education, marital status, race, place of birth, and BMI, the average mediation effects of HDL and TG on serum urate were 0.0059 (95% CI: 0.0029, 0.010; *p<* 2×10^–16^) and 0.0052 (95% CI: 0.0035, 0.010; *p<* 2×10^–16^), respectively. The mediated effects of HDL and TG accounted for the total effect of BMI on UA was 10.2% and 8.9%, respectively. There was no evidence suggesting that TC and LDL have mediated effects (**Table 2**).

**Table 2:** The results of observational mediation analysis.

	Estimation	95% CI lower	95% CI upper	*p*-value
TC ACME ADE Total effect Prop.Mediated HDL	−0.00031 0.058 0.058 −0.0046	−0.0010 0.050 0.049 −0.017	0.00 0.070 0.070 0.00	0.13 p<2 × 10^−16^ 2 × 10^−16^ 0.13
ACME	0.0059	0.0029	0.010	<2 × 10^−16^
ADE	0.052	0.045	0.060	<2 × 10^−16^
Total effect	0.058	0.051	0.060	<2 × 10^−16^
Prop.Mediated	0.102	0.049	0.15	<2 × 10^−16^
LDL				
ACME ADE Total effect Prop.Mediated	0.00013 0.058 0.058 0.0020	−0.00020 0.051 0.051 −0.0034	0.000 0.060 0.070 0.010	0.44 <2 × 10^−16^ <2 × 10^−16^ 0.44
TG ACME ADE Total effect Prop.Mediated	0.0052 0.053 0.058 0.089	0.0035 0.046 0.051 0.058	0.010 0.060 0.060 0.12	<2 × 10^−16^ <2 × 10^−16^ <2 × 10^−16^ <2 × 10^−16^

ACME, average causal mediation effect; ADE, average direct effect.

In the observational sensitivity analysis with 3,036 individuals, the results were consistent with the above, where the proportion of the mediated effects for HDL and TG was 10.7% and 5.1%. Moreover, there was also no evidence suggesting that LDL has a mediated effect (**Table 3**).

**Table 3:** The sensitivity of observational mediation analysis.

	Estimation	95% CI lower	95% CI upper	*p*-value
TC ACME ADE Total effect Prop.Mediated HDL	0.0002 0.058 0.058 0.0024	−0.00009 0.052 0.052 −0.0015	0.00 0.06 0.06 0.01	0.22 <2 × 10^−16^ <2 × 10^−16^ 0.22
ACME	0.0065	0.0041	0.010	<2 × 10^−16^
ADE	0.052	0.046	0.060	<2 × 10^−16^
Total effect	0.059	0.054	0.060	<2 × 10^−16^
Prop.Mediated	0.107	0.068	0.14	<2 × 10^−16^
LDL				
ACME ADE Total effect Prop.Mediated	0.00036 0.058 0.058 0.0066	−0.00025 0.052 0.052 −0.0043	0.000 0.060 0.060 0.020	0.26 <2 × 10^−16^ <2 × 10^−16^ 0.26
TG ACME ADE Total effect Prop.Mediated	0.0030 0.055 0.058 0.051	0.0015 0.050 0.052 0.025	0.00 0.060 0.060 0.080	<2 × 10^−16^ <2 × 10^−16^ <2 × 10^−16^ <2 × 10^−16^

ACME, average causal mediation effect; ADE, average direct effect.

### MR association of BMI with mediators and outcome

The two-sample MR showed that greater BMI was a risk factor for a higher level of serum urate. For each standard deviation of BMI increased, serum urate increased by 0.30 mg/dl (95% CI: 0.25, 0.34; *p* = 2.37×10^–35^). There was a causal relationship between BMI and the lipids. A greater BMI was associated with lower levels of HDL (*β* = −0.30, 95% CI: −0.32, −0.27; *p* = 1.39×10^–104^) and LDL (*β* = −0.20, 95% CI: −0.30, −0.098; *p* = 1.04×10^–4^) and a higher level of TG (*β* = 0.21, 95% CI: 0.18, 0.25; *p* = 1.15×10^–39.^). There was no evidence for a causal link between BMI and TC. The results of the sensitivity analysis are consistent (**Table 4**). **Table 5** shows the estimates of TC, HDL, LDL, and TG on serum urate. Among the four lipids, only TG has a causal relationship with serum urate (*β* = 0.22, 95% CI: 0.15, 0.29; *p* = 2.28×10^–10.^). For the relationship between HDL and serum urate, the intercept of MR-Egger was statistically significant. Thus, we adopted the MR-Egger’s result. Since TC, HDL, and LDL had no significant causal effect on serum urate, it indicated that only TG may be the mediator of BMI on serum urate.

**Table 4:** The effect of BMI on serum urate and lipid traits.

Outcome	Method	nSNPs	Betas (95% CI)	*p*-value
Urate	IVW	851	0.30 (0.25, 0.34)	2.37 × 10^−35^
	MR-Egger	851	0.34 (0.21, 0.48)	6.06 × 10^−7^
	Egger-intercept		−0.00076	0.45
	WME	851	0.31 (0.25, 0.36)	1.37 × 10^−32^
	MR-PRESSO	838	0.32 (0.29, 0.35)	6.71 × 10^−65^
TC	IVW	856	−0.030 (−0.075, 0.015)	0.18
	MR-Egger	856	−0.16 (−0.28, −0.042)	0.0071
	Egger-intercept		0.0022	0.018
	WME	856	−0.039 (−0.078, 0.00020)	0.054
	MR-PRESSO	820	−0.029 (−0.070, 0.012)	0.17
HDL	IVW	964	−0.30 (−0.32, −0.27)	1.39 × 10^−104^
	MR-Egger	964	−0.31 (−0.38, −0.23)	3.93 × 10^−14^
	Egger-intercept		0.00014	0.81
	WME	964	−0.29 (−0.31, −0.28)	5.18 × 10^−197^
	MR-PRESSO	863	−0.32 (−0.32, −0.30)	1.69 × 10^−203^
LDL	IVW	964	−0.068 (−0.10, −0.034)	9.45 × 10^−5^
	MR-Egger	964	−0.20 (−0.30, −0.098)	1.04 × 10^−4^
	Egger-intercept		0.0020	0.0066
	WME	964	−0.060 (−0.079, −0.042)	5.24 × 10^−10^
	MR-PRESSO	908	−0.049 (−0.054, −0.035)	4.40 × 10^−12^
TG	IVW	964	0.21 (0.18, 0.25)	1.15 × 10^−39^
	MR-Egger	964	0.18 (0.091, 0.28)	1.11 × 10^−4^
	Egger-intercept		0.00047	0.50
	WME	964	0.23 (0.21, 0.245)	5.62 × 10^−105^
	MR-PRESSO	853	0.25 (0.23, 0.27)	4.36 × 10^−142^

**Table 5:** Effect of lipid traits on serum urate.

Exposure	Method	nSNPs	Betas (95% CI)	*p*-value
TC	IVW	109	−0.035 (−0.096, 0.026)	0.26
	MR-Egger	109	0.019 (−0.10, 0.14)	0.75
	Egger-intercept		−0.0029	0.31
	WME	109	−0.048 (−0.097, 0.001)	0.055
	MR-PRESSO	101	−0.020 (−0.077, 0.037)	0.49
HDL	IVW	208	−0.090 (−0.14, −0.039)	0.00047
	MR-Egger	208	−0.00056 (−0.074, 0.072)	0.99
	Egger-intercept		−0.0032	0.0013
	WME	208	−0.059 (−0.12, 0.00069)	0.054
	MR-PRESSO	203	−0.080 (−0.13, −0.035)	0.00057
LDL	IVW	79	−0.0027 (−0.13, 0.12)	0.97
	MR-Egger	79	0.071 (−0.16, 0.30)	0.55
	Egger-intercept		−0.024	0.46
	WME	79	−0.043 (−0.14, 0.057)	0.39
	MR-PRESSO	74	−0.065 (−0.14, 0.0075)	0.084
TG	IVW	174	0.22 (0.15, 0.29)	2.28 × 10^−10^
	MR-Egger	174	0.11 (0.0024, 0.22)	4.66 × 10^−2^
	Egger-intercept		0.0033	0.013
	WME	174	0.15 (0.077, 0.23)	1.73 × 10^−4^
	MR-PRESSO	164	0.19 (0.14, 0.24)	1.84 × 10^−10^

**Figure 2:**
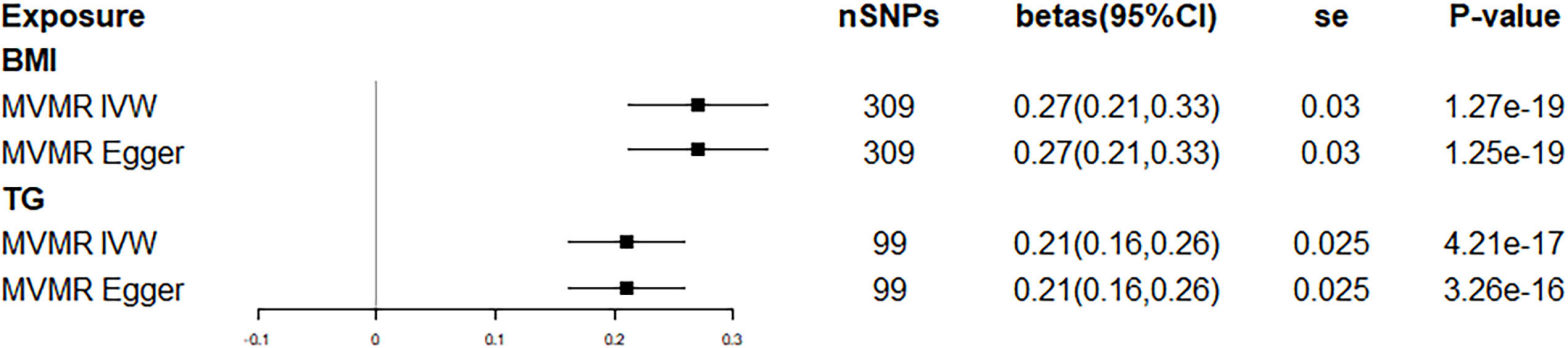
Forest plot of multivariable Mendelian randomization.

We used BMI and TG as independent variables in the multivariable Mendelian randomization model to estimate their direct effect on serum urate. The direct effects of BMI and TG on serum urate obtained by the IVW method were 0.27 (95% CI: 0.21, 0.33; *p* = 1.27×10^–19^) and 0.21 (95% CI: 0.16, 0.26, *p*=4.21×10^−17^), respectively; the direct effect estimated by MR-Egger was consistent with the IVW method (**Figure 2**). The mediated effect of TG was 0.045, the total effect of BMI on serum urate was 0.32, and the proportion of mediated effect of TG was 14.1%. Reverse MR analysis suggested that there was no reverse association between BMI, TC, HDL, LDL, TG, and serum urate (**Table 6**).

**Table 6:** The results of reverse MR.

	Methods	nSNPs	Betas (95% CI)	*p*-value
Urate–BMI	IVW	25	−0.022 (−0.054, 0.0092)	0.16
	MR-Egger	25		
	Slope		−0.031 (−0.085, 0.022)	0.26
	Intercept		0.00097	0.68
Urate–TC	IVW	33	0.031 (−0.038, 0.10)	0.38
	MR-Egger	33		
	Slope		0.070 (−0.040, 0.18)	0.22
	Intercept		−0.0045	0.38
Urate–TG	IVW	28	0.071 (−0.039, 0.18)	0.21
	MR-Egger	28		
	Slope		−0.0069 (−0.18, 0.16)	0.94
	Intercept		0.0010	0.26
Urate–HDL	IVW	28	−0.026 (−0.062, 0.010)	0.16
	MR-Egger	28		
	Slope		0.014 (−0.041, 0.068)	0.63
	Intercept		−0.0051	0.074
Urate–LDL	IVW	28	−0.0040 (−0.047, 0.039)	0.86
	MR-Egger	28		
	Slope		0.030 (−0.037, 0.097)	0.38
	Intercept		−0.0044	0.20

The Bonferroni method was used to correct the significance level of the causal association between exposures and serum urate, with p<0.0056 (0.05/9) being statistically significant.

## Discussion

In this study, observational and MR analyses supported that TG can explain the effect of BMI on serum urate to a certain extent. A potential mechanism is that TG synthesis accelerates the *de-novo* synthesis of ribose-5-phosphate to phosphoribosyl pyrophosphate through the common metabolic pathway of NADP–NADPH, resulting in increased serum urate production (34). In the observational analysis, we suggested that the mediating effect of TG accounted for 8.9% of the total effect (BMI-serum urate). For increasing the reliability of the results, we further took the two-step two-sample MR and multivariable Mendelian randomization to prove the mediation effect. Moreover, the two-sample MR showed that the proportion of mediation effect of TG was about 14.1%. Although the point estimates were different, both methods indicated the same conclusions. Moreover, existing studies have shown that greater BMI was associated with a higher level of TG and serum urate and the level of TG was also positively correlated with serum urate (12, 17, 19), which were consistent with our findings.

The point estimates of MR were slightly larger than the estimates of the observational study likely due to MR using genetic variants as instrumental variables to estimate the causal effect of exposure mediators and outcome. The influence of a gene on its phenotype has a lifetime effect. However, an observational study focuses on the effect of exposure on the outcome at a certain point in its investigation. These may explain the relatively large point estimates in MR. Additionally, this could be due to unknown confounding in the observational analysis. The studies by Alice R. Carter and others also suggested that the estimation of MR is greater than in observational studies (13, 35).

In the estimation of HDL-mediated effect, the results of the observational study showed that the proportion of HDL-mediated effect was 10.2%, while in MR, only the IVW approach suggested that serum urate decreased with the increase of HDL. The results of MR-Egger and WME showed that there was no causal effect between HDL and serum urate. Since the intercept term in the MR-Egger model was statistically significant, it indicated that the SNPs included in the MR model existed in horizontal pleiotropy, but the IVW method could not correct for the impact of the horizontal pleiotropy; for this, we adopted the result of the MR-Egger method here. The different results between observational analysis and MR may be due to the absence of unmeasured confounding in the multivariable linear regression model. In the analysis of LDL, observational and MR results suggested there was no causal relationship between LDL and serum urate. It suggested that LDL may not be the mediator in the pathway of BMI on serum urate.

In the two-sample MR analysis, there was some overlap between the samples of exposure and mediators, which could bias the estimates from the two-sample MR toward the confounded observational association, for example, weak instrument bias. Fortunately, bias from the sample overlap can be minimized by using strong instruments (*F* statistic greater than 10 for the instrument–exposure association) (36, 37). Moreover, the robust strength of our instruments for BMI (mean *F* statistic of 59) likely minimized any potential impact of sample overlap bias in our results.

In recent years, the incidence and prevalence of gout are increasing worldwide in close relation to the epidemic of obesity and metabolic syndrome. Therefore, it is very important to control BMI and its mediators on serum urate at the same time to reduce the risk of obesity-related gout. Our results suggested that reducing the level of TG may alleviate the increased prevalence of gout which is caused by the increasing BMI to a certain extent. More importantly, there is evidence indicating that statins can reduce the level of TG in our body (38); studies have also confirmed that there is a rare variant in APOC3 having a marked effect on TG levels, which provides a potential target for the development of drugs to reduce the level of TG (39, 40). This means that controlling BMI and TG simultaneously to reduce the occurrence of gout is a practical preventive measure.

### Strengths and limitations

This study used observational and two-sample MR analyses to determine the mediators and the size of the mediation effect. We have used these two methods, each with different potential sources of biases, to improve the reliability of our results through triangulation (41). For unmeasured confounding and reverse causality in observational analysis, we used MR to correct the biases. In MR analysis, we identified the mediators by using the two-step two-sample MR, and then multivariable Mendelian randomization was used to estimate the direct effect of exposure and mediator on outcome.

Both methods used in this study assumed that the relationship of BMI, TC, HDL, LDL, TG, and serum urate is linear, and whether there is a non-linear relationship needs to be further explored. Another important limitation of our study is that we assumed that there is no interaction between BMI and the four lipids. In view of the mediated effects, TG can only explain 14.1% of the effect of BMI on serum urate, indicating that there are still other factors that can explain the effect of BMI on serum urate, and these factors may be the future direction of follow-up research.

## Conclusions

In this study, observational and MR analyses were used to analyze the mediated effect of TC, HDL, LDL, and TG in the pathway of BMI on serum urate. Our results indicated that TG mediated the effect of BMI on serum urate to a certain extent, and the risk of gout may be reduced by controlling both BMI and TG.

## Data availability statement

Publicly available datasets were analyzed in this study. This data can be found here: https://www.cdc.gov/nchs/nhanes/index.htm https://gwas.mrcieu.ac.uk/.

## Ethics statement

Ethical review and approval was not required for the study on human participants in accordance with the local legislation and institutional requirements. Written informed consent for participation was not required for this study in accordance with the national legislation and the institutional requirements.

## Author contributions

TW had full access to all the data in the study and took responsibility for the integrity of the data and the accuracy of the data analysis. TW and LP designed the study. LP drafted the manuscript. LP and JJ performed the statistical analysis. TW, SH, JW, and XG critically revised the manuscript for important intellectual content. All authors contributed to the article and approved the submitted version.

## Funding

This study was supported by the National Natural Science Foundation of China (grant numbers 81872715, 82073674, 82103949); the Basic Research Project of Shanxi Province, China (grant number 20210302124186); and the Major Science and Technology Project of Shanxi Province (grant numbers: 202102130501003, 202005D121008).

## Acknowledgments

This work was made possible by the generous sharing of GWAS summary statistics and the observational data from NHANES. The authors acknowledge the use of summary-level data from the GIANT consortium, GUGC consortium, and UK Biobank consortium. We also thank the NHANES for offering individual data.

## Conflict of interest

The authors declare that the research was conducted in the absence of any commercial or financial relationships that could be construed as a potential conflict of interest.

## Publisher’s note

All claims expressed in this article are solely those of the authors and do not necessarily represent those of their affiliated organizations, or those of the publisher, the editors and the reviewers. Any product that may be evaluated in this article, or claim that may be made by its manufacturer, is not guaranteed or endorsed by the publisher.

## References

[1] Dalbeth N Choi HK Joosten LAB Khanna PP Matsuo H Perez-Ruiz F. Gout. Nat Rev Dis Primers (2019) 5(1):69. doi: 10.1038/s41572-019-0115-y 31558729

[2] Xia Y Wu Q Wang H Zhang S Jiang Y Gong T. Global, regional and national burden of gout, 1990-2017: A systematic analysis of the global burden of disease study. Rheumatol (Oxford) (2020) 59(7):1529–38. doi: 10.1093/rheumatology/kez476 31624843

[3] Smith E Hoy D Cross M Merriman TR Vos T Buchbinder R. The global burden of gout: Estimates from the global burden of disease 2010 study. Ann Rheum Dis (2014) 73(8):1470–6. doi: 10.1136/annrheumdis-2013-204647 24590182

[4] Dehlin M Jacobsson L Roddy E. Global epidemiology of gout: Prevalence, incidence, treatment patterns and risk factors. Nat Rev Rheumatol (2020) 16(7):380–90. doi: 10.1038/s41584-020-0441-1 32541923

[5] So A Thorens B. Uric acid transport and disease. J Clin Invest (2010) 120(6):1791–9. doi: 10.1172/JCI42344 PMC2877959 20516647

[6] Kuo C-F Grainge MJ Zhang W Doherty M. Global epidemiology of gout: Prevalence, incidence and risk factors. Nat Rev Rheumatol (2015) 11(11):649–62. doi: 10.1038/nrrheum.2015.91 26150127

[7] Tsushima Y Nishizawa H Tochino Y Nakatsuji H Sekimoto R Nagao H. Uric acid secretion from adipose tissue and its increase in obesity. J Biol Chem (2013) 288(38):27138–49. doi: 10.1074/jbc.M113.485094 PMC3779712 23913681

[8] Cheung KJ Tzameli I Pissios P Rovira I Gavrilova O Ohtsubo T. Xanthine oxidoreductase is a regulator of adipogenesis and PPARγ activity. Cell Metab (2007) 5(2):115–28. doi: 10.1016/j.cmet.2007.01.005 17276354

[9] Dalbeth N Merriman TR Stamp LK. Gout. Lancet (2016) 388(10055):2039–52. doi: 10.1016/s0140-6736(16)00346-9 27112094

[10] Dalbeth N Allan J Gamble GD Horne A Woodward OM Stamp LK. Effect of body mass index on serum urate and renal uric acid handling responses to an oral inosine load: Experimental intervention study in healthy volunteers. Arthritis Res Ther (2020) 22(1):259. doi: 10.1186/s13075-020-02357-y 33148335 PMC7641836

[11] Klop B Elte JW Cabezas MC. Dyslipidemia in obesity: Mechanisms and potential targets. Nutrients (2013) 5(4):1218–40. doi: 10.3390/nu5041218 PMC3705344 23584084

[12] Zhang Y Zhang M Yu X Wei F Chen C Zhang K. Association of hypertension and hypertriglyceridemia on incident hyperuricemia: An 8-year prospective cohort study. J Transl Med (2020) 18(1):409. doi: 10.1186/s12967-020-02590-8 33129322 PMC7603698

[13] Relton CL Davey Smith G. Two-step epigenetic mendelian randomization: A strategy for establishing the causal role of epigenetic processes in pathways to disease. Int J Epidemiol (2012) 41(1):161–76. doi: 10.1093/ije/dyr233 PMC3304531 22422451

[14] Burgess S Thompson SG. Multivariable mendelian randomization: The use of pleiotropic genetic variants to estimate causal effects. Am J Epidemiol (2015) 181(4):251–60. doi: 10.1093/aje/kwu283 PMC4325677 25632051

[15] Smith GD Ebrahim S. 'Mendelian randomization': Can genetic epidemiology contribute to understanding environmental determinants of disease? Int J Epidemiol (2003) 32(1):1–22. doi: 10.1093/ije/dyg070 12689998

[16] Gao X Wang H Wang T. Review on correction methods related to the pleiotropic effect in mendelian randomization. Chin J Epidemiol (2019) 40 (3):360–5 doi: 10.3760/cma.j.issn.0254-6450. 30884619

[17] Larsson SC Burgess S Michaelsson K. Genetic association between adiposity and gout: A mendelian randomization study. Rheumatol (Oxford) (2018) 57(12):2145–8. doi: 10.1093/rheumatology/key229 PMC6697177 30085130

[18] Xu L Borges MC Hemani G Lawlor DA. The role of glycaemic and lipid risk factors in mediating the effect of BMI on coronary heart disease: A two-step, two-sample mendelian randomisation study. Diabetologia (2017) 60(11):2210–20. doi: 10.1007/s00125-017-4396-y PMC6342872 28889241

[19] Yu X Wang T Huang S Zeng P. Evaluation of the causal effects of blood lipid levels on gout with summary level GWAS data: two-sample mendelian randomization and mediation analysis. J Hum Genet (2020) 66(5):465–73. doi: 10.1038/s10038-020-00863-0 33100326

[20] MacKinnon DP Lockwood CM Hoffman JM West SG Sheets V. A comparison of methods to test mediation and other intervening variable effects. psychol Methods (2002) 7(1):83–104. doi: 10.1037//1082-989x.7.1.83 11928892 PMC2819363

[21] Yengo L Sidorenko J Kemper KE Zheng Z Wood AR Weedon MN. Meta-analysis of genome-wide association studies for height and body mass index in approximately 700000 individuals of European ancestry. Hum Mol Genet (2018) 27(20):3641–9. doi: 10.1093/hmg/ddy271 PMC6488973 30124842

[22] Richardson TG Sanderson E Palmer TM Ala-Korpela M Ference BA Davey Smith G. Evaluating the relationship between circulating lipoprotein lipids and apolipoproteins with risk of coronary heart disease: A multivariable mendelian randomisation analysis. PloS Med (2020) 17(3):e1003062. doi: 10.1371/journal.pmed.1003062 32203549 PMC7089422

[23] Willer CJ Schmidt EM Sengupta S Peloso GM Gustafsson S Kanoni S. Discovery and refinement of loci associated with lipid levels. Nat Genet (2013) 45(11):1274–83. doi: 10.1038/ng.2797 PMC3838666 24097068

[24] Kottgen A Albrecht E Teumer A Vitart V Krumsiek J Hundertmark C. Genome-wide association analyses identify 18 new loci associated with serum urate concentrations. Nat Genet (2013) 45(2):145–54. doi: 10.1038/ng.2500 PMC3663712 23263486

[25] Assari S Nikahd A Malekahmadi MR Lankarani MM Zamanian H. Race by gender group differences in the protective effects of socioeconomic factors against sustained health problems across five domains. J Racial Ethn Health Disparities (2016) 4:884–94. doi: 10.1007/s40615-016-0291-3 27753050

[26] Masood M Aggarwal A Reidpath DD. Effect of national culture on BMI: A multilevel analysis of 53 countries. BMC Public Health (2019) 19(1):1212. doi: 10.1186/s12889-019-7536-0 31481044 PMC6719355

[27] Vishnu A Belbin GM Wojcik GL Bottinger EP Gignoux CR Kenny EE. The role of country of birth, and genetic and self-identified ancestry, in obesity susceptibility among African and Hispanic americans. Am J Clin Nutr (2019) 110(1):16–23. doi: 10.1093/ajcn/nqz098 31161206 PMC6599741

[28] Thompson JR Minelli C Del Greco MF. Mendelian randomization using public data from genetic consortia. Int J Biostat (2016) 12(2):1–11. doi: 10.1515/ijb-2015-0074 27092657

[29] Burgess S Butterworth A Thompson SG. Mendelian randomization analysis with multiple genetic variants using summarized data. Genet Epidemiol (2013) 37(7):658–65. doi: 10.1002/gepi.21758 PMC4377079 24114802

[30] Stekhoven DJ Buhlmann P. MissForest–non-parametric missing value imputation for mixed-type data. Bioinformatics (2012) 28(1):112–8. doi: 10.1093/bioinformatics/btr597 22039212

[31] Bowden J Davey Smith G Burgess S. Mendelian randomization with invalid instruments: effect estimation and bias detection through egger regression. Int J Epidemiol (2015) 44(2):512–25. doi: 10.1093/ije/dyv080 PMC4469799 26050253

[32] Bowden J Davey Smith G Haycock PC Burgess S. Consistent estimation in mendelian randomization with some invalid instruments using a weighted median estimator. Genet Epidemiol (2016) 40(4):304–14. doi: 10.1002/gepi.21965 PMC4849733 27061298

[33] Verbanck M Chen CY Neale B Do R. Detection of widespread horizontal pleiotropy in causal relationships inferred from mendelian randomization between complex traits and diseases. Nat Genet (2018) 50(5):693–8. doi: 10.1038/s41588-018-0099-7 PMC6083837 29686387

[34] Nejatinamini S Ataie-Jafari A Qorbani M Nikoohemat S Kelishadi R Asayesh H. Association between serum uric acid level and metabolic syndrome components. J Diabetes Metab Disord (2015) 14:70. doi: 10.1186/s40200-015-0200-z 26380228 PMC4570526

[35] Carter AR Gill D Davies NM Taylor AE Tillmann T Vaucher J. Understanding the consequences of education inequality on cardiovascular disease: Mendelian randomisation study. BMJ (2019) 365:l1855. doi: 10.1136/bmj.l1855 31122926 PMC6529852

[36] Pierce BL Burgess S. Efficient design for mendelian randomization studies: Subsample and 2-sample instrumental variable estimators. Am J Epidemiol (2013) 178(7):1177–84. doi: 10.1093/aje/kwt084 PMC3783091 23863760

[37] Burgess S Davies NM Thompson SG. Bias due to participant overlap in two-sample mendelian randomization. Genet Epidemiol (2016) 40(7):597–608. doi: 10.1002/gepi.21998 27625185 PMC5082560

[38] Wurtz P Wang Q Soininen P Kangas AJ Fatemifar G Tynkkynen T. Metabolomic profiling of statin use and genetic inhibition of HMG-CoA reductase. J Am Coll Cardiol (2016) 67(10):1200–10. doi: 10.1016/j.jacc.2015.12.060 PMC4783625 26965542

[39] Timpson NJ Walter K Min JL Tachmazidou I Malerba G Shin SY. A rare variant in APOC3 is associated with plasma triglyceride and VLDL levels in europeans. Nat Commun (2014) 5:4871. doi: 10.1038/ncomms5871 25225788 PMC4167609

[40] Drenos F Smith GD Ala-Korpela M. Metabolic characterization of a rare genetic variation within APOC3 and its lipoprotein lipase-independent effects. (2016) 9(3):231–9. doi: 10.1161/CIRCGENETICS.115.001302 PMC4920206 27114411

[41] Lawlor DA Tilling K Davey Smith G. Triangulation in aetiological epidemiology. Int J Epidemiol (2016) 45(6):1866–86. doi: 10.1093/ije/dyw314 PMC5841843 28108528

